# The association between SII and aging: evidence from NHANES 1999–2018

**DOI:** 10.3389/fpubh.2024.1418385

**Published:** 2024-06-27

**Authors:** Nanbu Wang, Lian Ren, Ziyuan Li, Yunhao Hu, Jingpei Zhou, Quan Sun, Bin Pei, Xinyu Li, Wanqing Peng, Jinyan Yu, Renhui Zhao, Ziting Huang, Zhenhu Chen, Guoxin Huang

**Affiliations:** ^1^State Key Laboratory of Traditional Chinese Medicine Syndrome, The First Affiliated Hospital of Guangzhou University of Chinese Medicine, Guangzhou, China; ^2^Center for Evidence-Based and Translational Medicine, Zhongnan Hospital of Wuhan University, Zhongnan Medical Journal Press, Zhongnan Hospital of Wuhan University, Wuhan, China; ^3^The First Clinical Medical College, Guangzhou University of Traditional Chinese Medicine, Guangzhou, China; ^4^Department of Evidence-Based Medicine Center, Xiangyang No.1 People’s Hospital, Hubei University of Medicine, Wuhan, China; ^5^Acupuncture and Rehabilitation Centre, The First Affiliated Hospital of Guangzhou University of Chinese Medicine, Guangzhou, China

**Keywords:** systemic immune-inflammatory index, aging, NHANES, phenotypic age, biological age

## Abstract

**Background:**

The study aimed to examine the association between the systemic immune-inflammation index (SII), a contemporary metric of systemic inflammatory response, and biological aging, which are closely interconnected processes.

**Methods:**

This cross-sectional study utilized 10 cycles of data from the NHANES database spanning from 1990 to 2018. The study examined the relationship between the SII index, calculated as P * N/L, where P represents preoperative peripheral platelet count, N represents neutrophil count, and L represents lymphocyte count, and biological aging. Biological aging was assessed through various methods, such as phenotypic age, phenotypic age acceleration (PhenoAgeAccel), biological age, and biological age acceleration (BioAgeAccel). Correlations were analyzed using weighted linear regression and subgroup analysis.

**Results:**

Among the 7,491 participants analyzed, the average age was 45.26 ± 0.34 years, with 52.16% being female. The average phenotypic and biological ages were 40.06 ± 0.36 and 45.89 ± 0.32 years, respectively. Following adjustment for potential confounders, elevated SII scores were linked to increased phenotypic age, biological age, Phenotypic age acceleration, and Biological age acceleration. Positive correlations were observed between health behavior and health factor scores and biological aging, with stronger associations seen for health factors. In health factor-specific analyses, the β coefficient was notably higher for high BMI. The robust positive associations between SII scores and both phenotypic age and biological age in the stratified analyses were consistently observed across all strata.

**Conclusion:**

The evidence from the NHANES data indicate that SII may serve as a valuable marker for assessing different facets of aging and health outcomes, such as mortality and the aging process. Additional research is warranted to comprehensively elucidate the implications of SII in the aging process and its utility as a clinical instrument for evaluating and addressing age-related ailments.

## 1 Introduction

Aging is characterized by systemic chronic inflammation, which is accompanied by cellular senescence, immunosenescence, organ dysfunction, and age-related diseases (1). Due to the increase in life expectancy and the decline in fertility rates, the global population is aging (2). Although everyone ages, the rate of aging is uneven, and the difference in aging rate between individuals is manifested by differences in susceptibility to death and disease (3). To further explain aging from the perspective of harmful inflammation and weakened immunity, inflammaging was introduced as an evolutionary perspective on immunosenescence, referring to the phenomenon of low-grade, chronic damage resulting from increased inflammation levels within the body (4). Later, inflammaging has been considered a hallmark of aging (5). Meanwhile, it is worth mentioning that can also damage the immune system, leading to immunosenescence during aging (6).

The systemic immune-inflammation index (SII) is an effective parameter to show the systemic immune and inflammation condition. It is a novel index that is used for the characterization of the severity of systemic inflammation. Recent studies have identified the high SII level as an independent predictor of poor outcomes in patients with multiple metabolic diseases (7). However, the association between the inflammatory level biomarker SII and aging is not well characterized.

Aging is a complex biological process that involves multiple dimensions of cells, tissues, and organs (8), so there are various ways to characterize biological aging, such as phenotypic age, biological age, leukocyte telomere length, and metabolic age score (9–11). In this study, phenotypic age and biological age were chosen to reflect aging. In general, phenotypic age corresponds to chronological age at the same risk of death, biological age refers to chronological age at the same physiological function (12). Phenotypic age and biological age calculated based on clinically observable data are considered to be more reliable predictors of aging outcomes.

Therefore, we examined the relationship between SII and aging in adults in this study, utilizing a large sample of people aged 20 to 80 years from the National Health and Nutrition Examination Survey (NHANES).

## 2 Methods

### 2.1 Study population

**Figure 1:**
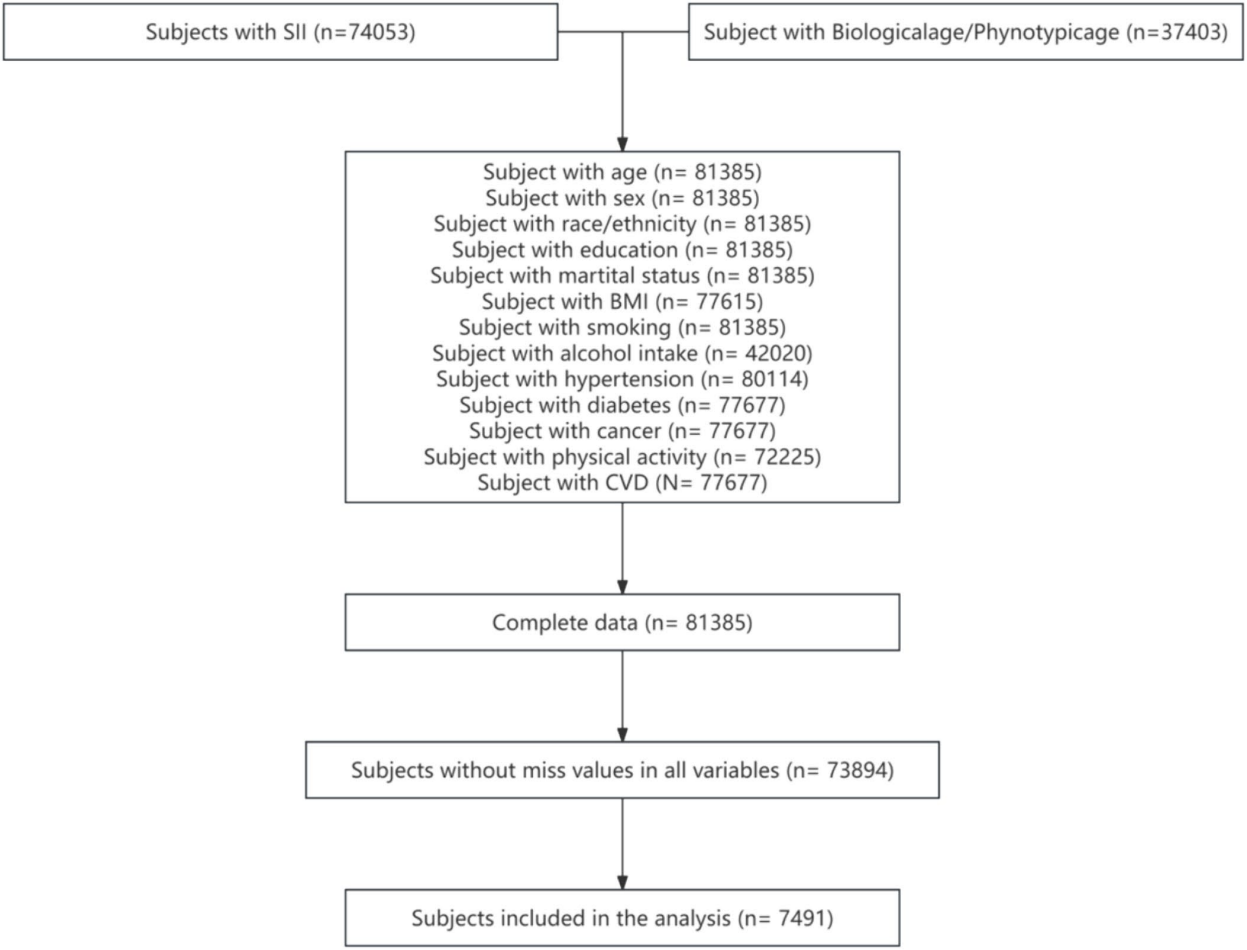
Flowchart of the participant selection from NHANES 1999–2018.

This study utilized 10 cycles of data spanning from 1990 to 2018 from the NHANES database. A total of 74,053 participants with SII data and 37,403 participants with phenotypic age and biological age data were extracted. The range of participants with covariate data was between 42,020 and 81,385. Following the combination of all variables and exclusion of participants with missing values, 7,491 participants were retained. Weighting was applied to represent 57,707,615 individuals. Further details can be found in Figure 1.

### 2.2 Measurement of SII

The calculation of the SII in this study is based on prior research conducted by Hu et al. (13). The formula for determining SII is SII = *P* * *N/L*, with *P* representing preoperative peripheral platelet count, *N* representing neutrophil count, and *L* representing lymphocyte count. Additionally, SII values are categorized into quartiles and subjected to a logarithmic transformation for normalization. In the division of quartiles, Q1 takes the 25% observation value after sorting the samples from small to large, which is [7.1505, 347.885]. Q2 takes the 50% observed value of the sample sorted from small to large, which is [347.885, 482]. Q3 takes the 75% observation value after sorting the samples from small to large, which is [482, 676]. Q4 takes the 100% observation value of the sample sorted from small to large, which is [676, 7290.9375].

### 2.3 Measurement of biological aging markers

Biological aging was assessed through the utilization of phenotypic age and biological age, each employing distinct biomarkers and calculation methodologies. Phenotypic age was calculated to consist of albumin, creatinine, glucose, Ln(C-reactive protein), lymphocyte percentage, mean cell volume, erythrocyte distribution width, alkaline ohosphatase, leukocyte count, chronological age (12). The Klemera and Doubal method was shown previously to be a reliable predictor of biological age and mortality that includes a set of eight biomarkers (Ln(C-reactive protein), serum creatinine, glycated hemoglobin, serum albumin, serum total cholesterol, serum urea nitrogen, serum alkaline phosphatase, and systolic blood pressure) (14, 15). Details of the calculation formulae are shown in Appendix 1.

Biological age was subtracted from chronological age (time elapsed since birth) to obtain biological age acceleration. Phenotypic age was subtracted from chronological age to obtain phenotypic age acceleration. Their values indicate a person appears older [positive value] or younger [negative value] than expected, physiologically.

### 2.4 Covariate assessment

In our study, covariates consisted of several factors previously displayed or assumed to be associated with SII and senescence. Covariables included age, sex, body mass index, race/ethnicity, marital status, education, smoking, alcohol intake, poverty, diabetes, hypertension, cerebrovascular disease (CVD), cancer, and physical activity.

### 2.5 Statistical analysis

This study utilized a multi-stage probability sampling approach from the NHANES database, with analysis conducted under the condition of sample weighting. The weights were adjusted according to the method outlined on the official NHANES website, using the weight variable “wtmec2yr” calculated as “1/10 * wtmec2yr.” Normality of the included variables was assessed using the Shapiro–Wilk test, with variables fitting a normal distribution described using mean and standard deviation. Differences among multiple groups were compared using ANOVA testing. When the variables deviated from a normal distribution, the median and number of quarterbacks were utilized to characterize the variables, and the Kruskal-Wallis test was employed to assess variances among multiple groups. Group differences were further examined using a Chi-square test.

Weighted linear regression was employed to investigate the association between aging acceleration and SII. The initial model utilized single-factor weighted liner regression, with aging acceleration as the independent variable and SII as the dependent variable. Model 1, which utilized single-factor weighted linear regression, employed aging acceleration as the independent variable and SII as the dependent variable. Model 2 was constructed, adjusting for the key covariates of age, sex, and race. Model 3 was developed by further adjusting for a comprehensive set of additional covariates, including age, sex, BMI, race/ethnicity, marital status, education, smoking habits, alcohol consumption, poverty level, diabetes status, hypertension, cardiovascular disease (CVD), cancer history, and physical activity.

Subgroup analysis was conducted based on various demographic and health-related variables including age, sex, BMI, race/ethnicity, marital status, education, smoking, alcohol intake, hypertension, CVD, cancer. The R (4.1.2) software was used for statistical analysis and drawing, and the “nhanesR” package was mainly used in the statistical analysis process of R (4.1.2) software.

## 3 Result

### 3.1 General characteristics of the study population

A total of 7,491 people were included in this study, of whom 47.84% were male and 52.16% were female, average age was 45.26 years and 76.07% were white. The clinical characteristics of the participants by SII quartiles are shown in Table 1, from which we can find statistically significant differences in sex, BMI, race/ethnicity, marital status, education, smoking, hypertension, physical activity, phenotypic age, biological age and phenotypic age acceleration (all *p* < 0.05).

**Table 1:** Weighted demographic characteristics of all participants.

Characteristics	Total	Q1	Q2	Q3	Q4	*p* value
Sex, %						< 0.0001
Female	52.16	58.75	54.39	52.77	43.67	
Male	47.84	41.25	45.61	47.23	56.33	
Age, mean (SD)	45.26(0.34)	44.26(0.51)	44.62(0.48)	45.36(0.50)	46.64(0.47)	< 0.001
Race/Ethnicity, %						< 0.0001
White	76.07	66.77	77.45	78.72	80.09	
Mexican	6.28	6.89	6.75	6.2	5.38	
Black	8.72	16.18	7.58	6.23	5.92	
Other	8.92	10.17	8.22	8.85	8.6	
BMI, mean (SD)	27.96(0.10)	27.15(0.19)	27.81(0.17)	28.13(0.16)	28.62(0.21)	< 0.0001
Marital status, %						< 0.0001
Married	60.64	59.02	62.89	62.69	57.75	
Living with partner	7.45	8.99	8.02	6.45	6.58	
Separated	2.27	1.79	2.11	2.18	2.91	
Divorced	4.14	3.75	3.72	3.48	5.56	
Widowed	9.05	7.8	7.12	8.71	12.35	
Never married	16.46	18.65	16.13	16.49	14.85	
Education, %						0.01
Under high school	14.44	15.41	14.24	13.6	14.65	
High school or equivalent	24.59	21.24	23.29	25.98	27.33	
Above high school	60.97	63.35	62.47	60.42	58.02	
Smoking, %						< 0.0001
Never	51.29	55.37	53.21	51.92	45.27	
Former	26.1	26.22	26.57	24.75	26.91	
Now	22.61	18.41	20.22	23.33	27.82	
Alcohol intake, %						0.22
Never	9.49	10.03	10.08	9.67	8.25	
Former	14.92	14.04	14.01	14.77	16.73	
Mild	37.64	37.94	39.31	38.37	35.01	
Moderate	17.02	17.92	16.66	16.56	17.06	
Heavy	20.93	20.07	19.94	20.62	22.95	
Poverty, mean (SD)	3.41 (1.82, 5.00)	3.40 (1.70, 5.00)	3.49 (1.93, 5.00)	3.57 (1.88, 5.00)	3.20 (1.74, 5.00)	0.01
Diabetes, %						0.07
No	94.13	94.55	94.44	94.76	92.84	
Yes	5.87	5.45	5.56	5.24	7.16	
Hypertension, %						0.01
No	67.31	70.95	68.69	66.24	63.93	
Yes	32.69	29.05	31.31	33.76	36.07	
CVD, %						0.07
No	93.3	93.8	92.95	94.23	92.27	
Yes	6.7	6.2	7.05	5.77	7.73	
Cancer, %						0.22
No	92.24	92.67	92.42	92.88	91.03	
Yes	7.76	7.33	7.58	7.12	8.97	
Physical activity(MET/Week), mean (SD)	756.00 (252.00, 2100.00)	910.00 (283.50, 2640.00)	756.00 (257.60, 1932.00)	720.00 (252.00, 1972.83)	720.00 (252.00, 1897.47)	< 0.001
Phenotypic Age, mean (SD)	40.06 (0.36)	36.55 (0.57)	38.37 (0.50)	40.26 (0.45)	44.52 (0.54)	< 0.0001
Biological Age, mean (SD)	45.89 (0.32)	44.77 (0.49)	45.37 (0.43)	45.94 (0.48)	47.30 (0.45)	< 0.001
Phenotypic age acceleration, mean (SD)	−5.20 (0.11)	−7.70 (0.17)	−6.24 (0.15)	−5.11 (0.16)	−2.12 (0.20)	< 0.0001
Biological age acceleration, mean (SD)	0.63 (0.08)	0.52 (0.13)	0.75 (0.14)	0.58 (0.15)	0.66 (0.14)	0.57

Q1 represents the range of SII within [7.1505, 347.885]. Q2 represents the range of SII within [347.885, 482]. Q3 represents the range of SII within [482, 676]. Q4 represents the range of SII within [676, 7290.9375].

### 3.2 Relationship between SII and aging for phenotypic age acceleration

After performing a weighted multivariate linear regression analysis (Table 2). Following the non-normal transformation of SII, the linear regression analysis revealed a statistically significant positive association between log (SII) and phenotypic age acceleration. Specifically, the estimated beta coefficient (95% confidence interval) was 3.89 (3.49, 4.28) in model 1, with a *p*-value of less than 0.0001. Subsequent models yielded beta coefficients of 4.34 (3.94, 4.75) in model 2 and 3.7 (3.32, 4.09) in the fully adjusted model 3, all with *p*-values less than 0.0001. Sensitivity analysis involved transforming SII into quartiles, with the highest quartile showing a beta coefficient of 5.58 (5.10, 6.06) compared to the lowest quartile in model1. In comparison to the lowest quartile (model 1), the results demonstrated statistically significant differences in β values for models 2 and 3, with respective values of 6.22 (95% CI 5.75, 6.69) and 5.28 (95% CI 4.85, 5.71), both yielding a *p* value of less than 0.0001.

**Table 2:** Weighted multivariate linear analysis SII and senescence for phenotypic age acceleration.

Characteristics	Model 1		Model 2		Model 3	
	β (95% CI)	*p* value	β (95% CI)	*p* value	β (95% CI)	*p* value
Log (SII)	3.89 (3.49, 4.28)	<0.0001	4.34 (3.94, 4.75)	<0.0001	3.7 (3.32, 4.09)	<0.0001
Stratified by SII quartiles						
Q1	1.00 (1.00, 1.00)		1.00 (1.00, 1.00)		1.00 (1.00, 1.00)	
Q2	1.46 (1.02, 1.90)	<0.0001	1.87 (1.44, 2.30)	<0.0001	1.55 (1.15, 1.94)	<0.0001
Q3	2.59 (2.14, 3.05)	<0.0001	3.07 (2.61, 3.53)	<0.0001	2.61 (2.20, 3.01)	<0.0001
Q4	5.58 (5.10, 6.06)	<0.0001	6.22 (5.75, 6.69)	<0.0001	5.28 (4.85, 5.71)	<0.0001

Model 1: unadjusted mode; Model 2: adjusted for age, sex, race; Model 3: additionally adjusted for age, sex, BMI, race/ethnicity, marital status, education, smoking, alcohol intake, poverty, diabetes, hypertension, CVD, cancer, and physical activity. Q1 represents the range of SII within [7.1505, 347.885]. Q2 represents the range of SII within [347.885, 482]. Q3 represents the range of SII within [482, 676]. Q4 represents the range of SII within [676, 7290.9375].

### 3.3 Relationship between SII and aging for biological age acceleration

After performing a weighted multivariate linear regression analysis (Table 3). Following the non-normal transformation of SII, the linear regression analysis revealed a statistically significant positive association between log(SII) and biological age acceleration. Specifically, in model 1, the estimated beta coefficient (β) was 0.15 (95% confidence interval [CI]: −0.06, 0.35) with a corresponding *p*-value of 0.15. In model 2, the association strengthened with a β of 0.52 (95% CI: 0.30, 0.73) and a p-value of less than 0.0001. Finally, in the fully adjusted model 3, the estimated β was 0.33 (95% CI: 0.13, 0.53) with a p-value of less than 0.0001. The Sensitivity Index of the study was converted from a continuous variable to a categorical variable (quartiles) for further analysis, as shown in Table 2. When compared to the lowest quartile, the results for model 1 yielded a β coefficient of 0.14 (95% CI: −0.19, 0.47) with a *p* value of 0.39. In model 2, the β coefficient was 0.67 (95% CI: 0.33, 1.02) with a *p* value of less than 0.0001. Model 3 showed a β coefficient of 0.37 (95% CI, 0.05, 0.70) with a *p* value of 0.02.

**Table 3:** Weighted multivariate linear analysis SII and senescence for biological age acceleration.

Characteristics	Model 1		Model 2		Model 3	
	β (95% CI)	*p* value	β(95% CI)	*p* value	β(95% CI)	*p* value
Log(SII)	0.15 (−0.06, 0.35)	0.15	0.52 (0.30, 0.73)	<0.0001	0.33 (0.13, 0.53)	<0.002
Stratified by SII quartiles						
Q1	1.00 (1.00, 1.00)		1.00 (1.00, 1.00)		1.00 (1.00, 1.00)	
Q2	0.24 (−0.11, 0.59)	0.01	0.43 (0.10, 0.76)	0.01	0.25 (−0.07, 0.58)	0.12
Q3	0.06 (−0.28, 0.40)	0.04	0.35 (0.02, 0.69)	0.04	0.15 (−0.17, 0.47)	0.35
Q4	0.14 (−0.19, 0.47)	0.39	0.67 (0.33, 1.02)	<0.001	0.37 (0.05, 0.70)	0.02

Model 1: unadjusted mode; Model 2: adjusted for age, sex, race; Model 3: additionally adjusted for age, sex, BMI, race/ethnicity, marital status, education, smoking, alcohol intake, poverty, diabetes, hypertension, CVD, cancer, and physical activity. Q1 represents the range of SII within [7.1505, 347.885]. Q2 represents the range of SII within [347.885, 482]. Q3 represents the range of SII within [482, 676]. Q4 represents the range of SII within [676, 7290.9375].

### 3.4 Subgroup analysis

We found that the risk of aging was not consistently associated with increased log(SII) levels (Table 4) in some subgroups. Overall, for both phenotypic age acceleration and biological age acceleration, sex, marital status, education, smoke, alcohol intake, hypertension, and CVD was statistically significant (*p* > 0.05).

**Table 4:** Subgroup analysis for the association between SII and senescence.

Character	Phenotypic age acceleration		Biological age acceleration	
	β (95% CI)	*p*-value	β (95% CI)	*p*-value
Sex				
Male	3.22 (2.56, 3.88)	<0.0001	0.54 (0.18, 0.89)	0.004
Female	4.09 (3.59, 4.59)	<0.0001	0.18 (−0.09, 0.44)	0.18
Age				
= < 60	4.08 (3.67, 4.49)	<0.0001	0.31 (0.05, 0.58)	0.02
60–80	2.2 (1.03, 3.36)	<0.001	0.23 (−0.21, 0.67)	0.3
> = 80	2.21 (−0.07, 4.49)	0.06	0.66 (−0.89, 2.22)	0.39
BMI				
Low	3.2 (2.54, 3.86)	<0.0001	0.23 (−0.08, 0.54)	0.14
Normal	3.81 (3.14, 4.48)	<0.0001	0.4 (−0.04, 0.83)	0.07
High	4.55 (3.95, 5.15)	<0.0001	0.52 (0.13, 0.90)	0.01
Race/Ethnicity				
White	3.41 (2.97, 3.86)	<0.0001	0.33 (0.08, 0.57)	0.01
Mexican	4.14 (3.63, 4.66)	<0.0001	0.51 (−0.06, 1.08)	0.08
Black	4.45 (3.52, 5.38)	<0.0001	0.47 (−0.05, 0.99)	0.07
Other	4.99 (3.79, 6.19)	<0.0001	0.49 (−0.46, 1.43)	0.31
Marital status				
Married	3.66 (3.16, 4.17)	<0.0001	0.14 (−0.14, 0.41)	0.32
Living with partner	3.36 (2.61, 4.11)	<0.0001	0.69 (0.04, 1.34)	0.04
Separated	4.04 (2.46, 5.62)	<0.0001	−0.36 (−1.54, 0.82)	0.53
Divorced	4.7 (3.72, 5.69)	<0.0001	0.7 (−0.09, 1.48)	0.08
Widowed	2.79 (1.31, 4.26)	<0.001	0.14 (−0.85, 1.12)	0.78
Never married	3.49 (2.38, 4.60)	<0.0001	0.67 (0.17, 1.17)	0.01
Education				
Under high school	3.74 (3.05, 4.44)	<0.0001	0.1 (−0.41, 0.60)	0.71
High school or equivalent	3.91 (3.26, 4.56)	<0.0001	0.46 (−0.03, 0.95)	0.06
Above high school	3.58 (3.05, 4.10)	<0.0001	0.34 (0.06, 0.62)	0.02
Smoking				
Never	3.82 (3.38, 4.27)	<0.0001	0.32 (0.02, 0.63)	0.04
Former	3 (2.00, 4.00)	<0.0001	0.23 (−0.18, 0.64)	0.27
Now	4.14 (3.29, 4.98)	<0.0001	0.48 (0.01, 0.95)	0.05
Alcohol intake				
Never	4.75 (3.67, 5.82)	<0.0001	0.39 (−0.48, 1.26)	0.37
Former	2.99 (1.17, 4.80)	0.002	0.49 (−0.04, 1.02)	0.07
Mild	3.69 (3.24, 4.15)	<0.0001	0.16 (−0.18, 0.50)	0.35
Moderate	3.64 (2.99, 4.30)	<0.0001	0.34 (−0.04, 0.72)	0.08
Heavy	3.82 (3.19, 4.45)	<0.0001	0.33 (−0.20, 0.87)	0.22
Hypertension				
No	3.7 (3.20, 4.20)	<0.0001	0.25 (0.01, 0.49)	0.04
Yes	3.65 (2.98, 4.31)	<0.0001	0.42 (0.02, 0.83)	0.04
Cancer				
No	3.99 (3.63, 4.36)	<0.0001	0.34 (0.12, 0.57)	0.003
Yes	1.28 (−0.89, 3.44)	0.24	0.29 (−0.31, 0.89)	0.34
CVD				
No	3.71 (3.30, 4.13)	<0.0001	0.31 (0.11, 0.52)	0.004
Yes	3.29 (1.79, 4.79)	<0.0001	0.29 (−0.62, 1.20)	0.53

## 4 Discussion

To elucidate the relationship between SII and biological aging, we carried out a cross-sectional analysis of 7,491 participants from the NHANES cohort. Affirmative associations were revealed between SII and its health behavior and health factor subscales and biological aging. Stratified analyses illustrated that the relationship between SII and biological aging remained stable across stratification factors.

Biological aging is known to be assessed in a variety of ways, such as phenotypic age, biological age, white blood cell telomere length, and metabolic age score. Therefore, several studies have evaluated the relationship between SII and biological aging assessed using other modalities. A previous study using the NHANES database from 1999 to 2018 found a positive correlation, there is a positive correlation between SII and rheumatoid arthritis. This study also found that SII is a novel, valuable, and convenient inflammatory marker that can be used to predict the risk of rheumatoid arthritis in US adults (16). Xia et al. discovered that among participants with all-cause mortality and cardiovascular mortality, SII was closely associated with cardiovascular death and all-cause death, and more attention should be paid to systemic inflammation to provide better preventive strategies (16).

It is worth noting that our results indicate that positive correlations were observed between health behavior and health factor scores and biological aging, with stronger associations seen for health factors. Various factors have been found to exert similar influences on both Phenotypic age and Biological age. Health-related factors, such as abstaining from alcohol and tobacco, having a favorable marital and educational status, maintaining normal blood pressure, and having no history of cardiovascular disease, have been shown to decelerate the aging process. Numerous studies have provided evidence that a normal body mass index (BMI) can extend lifespan, enhance healthy life expectancy, promote better physical functioning among older individuals, and impede the aging process (17, 18). Restoring aberrant BMI is a novel and promising strategy to combat aging. Moreover, different racial backgrounds have been found to impact indicators of aging and the duration of a healthy lifespan in healthy adults through their effects on multiple molecular pathways and cell types. Blood pressure is commonly regarded as a significant modifiable vascular risk factor in the prevention or postponement of aging and dementia (19, 20). Aging is distinguished by changes in neuro-cardiovascular regulatory mechanisms, resulting in compromised patterns of physiological variability (21). The alterations observed in both aging and blood pressure involve numerous shared molecular mechanisms, such as subclinical inflammation, heightened production of reactive oxygen species, impaired endothelial function, increased arterial stiffness, autonomic dysfunction, genomic instability, oxidative damage to mitochondria, and epigenetic modifications, among other factors (21, 22). Furthermore, it has been observed that hypertension is linked to cortical atrophy, specifically in the hippocampus and frontal cortical regions, thereby expediting the process of brain aging (23). Our study reveals that health factors exhibit a stronger correlation with aging compared to health behaviors, likely due to the direct association between health factors and the aging process, while health behaviors mitigate aging by enhancing health factors.

Diseases associated with aging, including osteoporosis, malignant tumors, arteriosclerosis, Parkinson’s disease, and Alzheimer’s disease, now also affect younger populations (24). Jia et al. reported a 19-year-old Chinese youth with probable AD (25). Stroke and other age-related diseases are also showing a trend toward youthfulness (24). The average age of 7,491 participants in this study is 45.26 ± 0.34 years old, which belongs to the middle-aged age range. Unlike the older adult, patients aged 40–54 are the most productive and responsible age group, the main source of income, and often the parents of young children (26). The early onset of age-related diseases is becoming a major medical and social issue (27). Valuable indicators for evaluating aging and health outcomes are particularly important, and we look forward to more research on SII and aging in different age groups in the future, in order to comprehensively elucidate the significance of SII in the aging process and its effectiveness as a clinical tool for evaluating and addressing age-related diseases.

The current investigation possesses various notable attributes. Firstly, the NHANES employed a sophisticated multistage probability sampling design, ensuring the selection of a representative sample from the civilian non-institutionalized population. Consequently, the extrapolation of our results to the entire US civilian noninstitutionalized population can be deemed highly reliable. Secondly, this study employed stratified analyses to investigate the relationship between SII and biological aging across diverse populations, thereby enhancing the generalizability of the findings. Overall, these results have significant implications for public health in terms of aging prevention.

It is imperative to recognize the constraints of the present study. Primarily, the cross-sectional design hinders the ability to establish a causal association between SII and biological aging. Consequently, forthcoming investigations should utilize prospective methodologies to substantiate the effectiveness of the SII. Moreover, the assessment of health behavior indicators in this study was reliant on self-reported questionnaires, susceptible to recall bias. Furthermore, the analysis of biological aging was constrained to clinical indicators such as phenotypic age and biological age, with no exploration of molecular or cellular components. Nonetheless, employing two separate methodologies for assessing biological aging enabled the reciprocal confirmation of our results, thereby greatly bolstering the robustness and credibility of our conclusions.

## 5 Conclusion

This study examined a demographically diverse sample of adults in the United States and found significant positive associations between the systemic immune inflammation index (SII), health behavior scores, health factor scores, and biological aging. Of note, health factors showed a stronger positive correlation with biological aging compared to health behaviors, with blood glucose and blood pressure being particularly influential among the health factors. These findings emphasize the potential effectiveness of SII in hastening the aging process and reducing biological aging. Additionally, they underscore the significance of embracing a healthy lifestyle, which plays a crucial role in averting aging.

## Data availability statement

The original contributions presented in the study are included in the article/Supplementary material, further inquiries can be directed to the corresponding author.

## Ethics statement

This study was based on publicly available datasets. Ethical review and approval was not required for the study, in accordance with the local legislation and institutional requirements. For the NHANES data studied, ethics approval and consent to participate in NHANES was approved by the National Center for Health Statistics Research Ethics Review Board.

## Author contributions

NW: Conceptualization, Data curation, Formal analysis, Funding acquisition, Investigation, Methodology, Project administration, Resources, Software, Supervision, Validation, Visualization, Writing – original draft, Writing – review & editing. LR: Conceptualization, Data curation, Formal analysis, Funding acquisition, Investigation, Methodology, Project administration, Resources, Software, Supervision, Validation, Visualization, Writing – original draft, Writing – review & editing. ZL: Conceptualization, Data curation, Formal analysis, Funding acquisition, Investigation, Methodology, Project administration, Resources, Software, Supervision, Validation, Visualization, Writing – original draft, Writing – review & editing. YH: Conceptualization, Writing – review & editing. JZ: Data curation, Writing – review & editing. QS: Formal analysis, Writing – review & editing. BP: Funding acquisition, Writing – review & editing. XL: Investigation, Writing – review & editing. WP: Methodology, Writing – review & editing. JY: Project administration, Writing – review & editing. RZ: Resources, Writing – review & editing. ZH: Software, Writing – review & editing. ZC: Supervision, Writing – review & editing. GH: Software, Writing – review & editing.
