# Supplementary material for: The association between SII and aging: evidence from NHANES 1999–2018
Source: Front Public Health. 2024 Jun 27;12:1418385. doi: 10.3389/fpubh.2024.1418385 (PMC11236748; doi:10.3389/fpubh.2024.1418385)
Supplement: Supplementary file 1 [file Data_Sheet_1.docx]

Supplementary Material

# Formulae for biological age and phenotypic age.

Phenotypic age was determined through the application of the subsequent formula, in which, xb = −19.907−0.0336×Albumin + 0.0095×Creatinine + 0.1953×Glucose + 0.0954×LnCRP−0.0120×Lymphocyte Percentage + 0.0268×Mean Cell Volume + 0.3306 × Erythrocyte Distribution Width + 0.00188 ×Alkaline Phosphatase + 0.0554×Leukocyte Count + 0.0804 ×chronological age.

$$\text{Phenotypic age }=141.50+\frac{Ln\left[-0.00553\times L\text{n}\left(\text{exp}\left(\frac{\left(\text{-1.51714}\times\text{exp}\left(\text{xb}\right)\right)}{0.0076927}\right)\right)\right]}{0.09165}$$

Klemera introduced a method for determining biological age using a set of eight biomarkers (Ln-CRP, serum creatinine, glycated hemoglobin, serum albumin, serum total cholesterol, serum urea nitrogen, serum alkaline phosphatase, and systolic blood pressure). The variables j and i represent the number of biomarkers and samples, respectively. The parameters k, q, and s denote the slope, intercept, and root mean square error of the regression analysis between biomarkers and chronological age.The variance explained by the regression of biomarkers against chronological age is plotted as r_j_^2^.
